# SARS-CoV-2 exposure in Malawian blood donors: an analysis of seroprevalence and variant dynamics between January 2020 and July 2021

**DOI:** 10.1186/s12916-021-02187-y

**Published:** 2021-11-19

**Authors:** Jonathan Mandolo, Jacquline Msefula, Marc Y. R. Henrion, Comfort Brown, Brewster Moyo, Aubrey Samon, Thandeka Moyo-Gwete, Zanele Makhado, Frances Ayres, Thopisang Motlou, Nonkululeko Mzindle, Newton Kalata, Adamson S. Muula, Gaurav Kwatra, Natasha Nsamala, Andrew Likaka, Thom Mfune, Penny L. Moore, Bridon Mbaya, Neil French, Robert S. Heyderman, Todd Swarthout, Kondwani C. Jambo

**Affiliations:** 1 Malawi-Liverpool-Wellcome Trust Clinical Research Programme (MLW), Blantyre, Malawi; 2 Liverpool School of Tropical Medicine, Liverpool, UK; 3 Malawi Blood Transfusion Services (MBTS), Blantyre, Malawi; 4 National Institute for Communicable Diseases of the National Health Laboratory Services, Johannesburg, South Africa; 5 MRC Antibody Research Unit, School of Pathology, University of the Witwatersrand, Johannesburg, South Africa; 6 Kamuzu University of Health Sciences (KUHes), Blantyre, Malawi; 7 Respiratory and Meningeal Pathogens Research Unit, Johannesburg, South Africa; 8 Department of Science/National Research Foundation: Vaccine Preventable Diseases, Faculty of Health Science, University of the Witwatersrand, Johannesburg, South Africa; 9 Department of Clinical Microbiology, Christian Medical College, Vellore, India; 10 LIKA-UFPE, Universidade Federal de Pernambuco, Recife, Brazil; 11 University of Liverpool, Liverpool, UK; 12 NIHR Global Health Research Unit on Mucosal Pathogens, Research Department of Infection, Division of Infection and Immunity, University College London, London, UK

**Keywords:** SARS-CoV-2, Seroprevalence, Malawi, Blood donors

## Abstract

**Background:**

By August 2021, the COVID-19 pandemic has been less severe in sub-Saharan Africa than elsewhere. In Malawi, there have been three subsequent epidemic waves. We therefore aimed to describe the dynamics of SARS-CoV-2 exposure in Malawi.

**Methods:**

We measured the seroprevalence of anti-SARS-CoV-2 antibodies amongst randomly selected blood transfusion donor sera in Malawi from January 2020 to July 2021 using a cross-sectional study design. In a subset, we also assessed in vitro neutralisation against the original variant (D614G WT) and the Beta variant.

**Results:**

A total of 5085 samples were selected from the blood donor database, of which 4075 (80.1%) were aged 20–49 years. Of the total, 1401 were seropositive. After adjustment for assay characteristics and applying population weights, seropositivity reached peaks in October 2020 (18.5%) and May 2021 (64.9%) reflecting the first two epidemic waves. Unlike the first wave, both urban and rural areas had high seropositivity in the second wave, Balaka (rural, 66.2%, April 2021), Blantyre (urban, 75.6%, May 2021), Lilongwe (urban, 78.0%, May 2021), and Mzuzu (urban, 74.6%, April 2021). Blantyre and Mzuzu also show indications of the start of a third pandemic wave with seroprevalence picking up again in July 2021 (Blantyre, 81.7%; Mzuzu, 71.0%). More first wave sera showed in vitro neutralisation activity against the original variant (78% [7/9]) than the beta variant (22% [2/9]), while more second wave sera showed neutralisation activity against the beta variant (75% [12/16]) than the original variant (63% [10/16]).

**Conclusion:**

The findings confirm extensive SARS-CoV-2 exposure in Malawi over two epidemic waves with likely poor cross-protection to reinfection from the first on the second wave. The dynamics of SARS-CoV-2 exposure will therefore need to be taken into account in the formulation of the COVID-19 vaccination policy in Malawi and across the region. Future studies should use an adequate sample size for the assessment of neutralisation activity across a panel of SARS-CoV-2 variants of concern/interest to estimate community immunity.

**Supplementary Information:**

The online version contains supplementary material available at 10.1186/s12916-021-02187-y.

## Background

As of 19 August 2021, more than 209 million people globally have been infected with the severe acute respiratory syndrome (SARS-CoV-2), resulting in more than 4.39 million deaths [1, 2]. The potential risk from SARS-CoV-2 to Africa was identified early in the global pandemic [3]. As the epicentre of transmission moved from East Asia to West Asia, to Europe, and then to North America, there was speculation as to the likely impact of the pandemic on the African continent with its high rates of infectious diseases including HIV, poverty, and undernutrition, as well as a fragile healthcare system [3, 4]. However, so far, evidence has shown that the epidemiology of the COVID-19 pandemic in sub-Saharan Africa has been different from the Americas, China, and Europe [1]. Available seroprevalence data indicate that SARS-CoV-2 has circulated more widely in African populations than can be deduced from the number of reported confirmed cases, hospitalisations, and deaths [5–8]. This has led to speculations that SARS-CoV-2 could have circulated longer in sub-Saharan Africa than the first confirmed cases, but current evidence is limited. Others have speculated that a high prevalence of circulating seasonal coronaviruses could have induced some cross-reactive protective immunity [9].

In Malawi, though there were initial plans by the government in April 2020 to implement a national lockdown of social and commercial activities, these were never implemented [10]. As such, unlike other countries in the region, there were no systematic lockdowns but only some curfew restrictions. Schools were officially closed (from 23 March 2020 to 7 September 2020), and many social gathering settings (including restaurants and places of worship) voluntarily closed or had significantly reduced services as a requirement from the government [11]. Malawi closed its borders and airports from April 2020 to September 2020, with limited essential traffic allowed in and out of the country [12]. As of 18 August 2021, 58,861 people were confirmed to have been infected by SARS-CoV-2 in Malawi, resulting in 2012 deaths, and 265,491 people had been fully vaccinated with either the AstraZeneca (AZD1222) or Jansen vaccines [1]. However, due to limited testing, the true burden of COVID-19 in Malawi remains unclear.

Blood donor serosurveys are well-used surrogates for population surveillance of infectious diseases and have been used together with healthcare worker serosurveys to estimate and monitor the extent of the SARS-CoV-2 pandemic in several countries [5–8]. Here, we report results of a national serosurvey using archived serum samples from blood transfusion donors across Malawi from January 2020 to July 2021, generated using a World Health Organization (WHO)-recommended anti-SARS-CoV-2 receptor-binding domain total antibody assay with high sensitivity and specificity, supported by SARS-CoV-2 pseudovirus neutralisation assays to explore variant specificity. We aimed to estimate the magnitude of SARS-CoV-2 population exposure, describe the kinetics of the exposure, approximate the period of SARS-CoV-2 introduction into the population, and determine the viral variants responsible for the epidemic waves.

## Methods

### Study setting and population

The Malawi Blood Transfusion Service (MBTS) maintains an archive of sera from all blood donations received in their Malawi facilities, namely Blantyre (Odala), Balaka, Lilongwe, and Mzuzu (average of 70,000 annually before the COVID-19 pandemic). All blood donors undergo routine screening through a self-administered questionnaire, one-on-one assessment, and a mini-health screening by MBTS staff. Donors meeting the routine donor ineligibility criteria including age below 15 or above 65 years; haemoglobin below 12.5 g/dl; past medical history suggestive of human immunodeficiency virus (HIV), hepatitis, or syphilis; and past or present history of renal, cardiovascular, central nervous system, and metabolic disorders [13] were excluded. All donated samples are screened for transfusion transmissible infections (TTI, including HIV). The sera are archived at − 80 °C and retained by MBTS for up to 5 years for retrospective analysis purposes. MBTS blood donation services include both static and mobile sites.

### Sample selection and processing

Using the MBTS sample archive database, we randomly selected (using STATA’s gsample command) sera collected from HIV-seronegative individuals aged 15–65 years old, which is the allowable age for blood donation in Malawi. Parameters including sex, age, location, and time were also extracted from the database for analysis. For Mzuzu, Lilongwe, and Blantyre, selected samples were only from static urban donation sites. For Balaka, a largely rural district with smaller total donations, selected samples included donations from both static and mobile sites, including semi-rural and rural sites. The sites covered all four defined regions of Malawi: Mzuzu is the capital of the northern region and the third largest city, by population, in Malawi (population of 221,272). Lilongwe is Malawi’s capital city located in the central region (population of 989,318). Blantyre is the capital of Malawi’s southern region (population of 800,264). Balaka is a rural district of 438,379 residents, located in the eastern region. All population data are from the 2018 Malawi Population and Housing Census [14].

### Measurement of SARS-CoV-2 antibodies by enzyme-linked immunosorbent assay (ELISA)

#### SARS-CoV-2 receptor-binding domain (RBD) total antibody ELISA

We used the WHO-recommended WANTAI SARS-CoV-2 Ab commercial ELISA kit to detect total SARS-CoV-2 antibodies, following the manufacturer’s instructions (Beijing Wantai Biological Pharmacy Enterprise Co., Ltd., China; WS-1096). The sensitivity and specificity of the assay as independently validated are 97% [*95% CI* 83.3 to 99.4] and 98% [91.3 to 99.3], respectively [15]. Specimens giving a ratio of < 0.9 were reported as negative for this assay, a ratio of > 1.1 were reported as positive, and a ratio between 0.9 and 1.1 were reported as borderline. Samples with borderline results were retested using a confirmatory assay described below.

#### Confirmatory SARS-CoV-2 spike 2 and nucleoprotein immunoglobulin G (IgG) antibody ELISA

The COVID-19 IgG RUO commercial ELISA kit (Omega diagnostics, UK) uses 96-well microtitre plates pre-coated with purified SARS-CoV-2 spike (S2) and nucleoprotein (N) antigens to detect anti-SARS-CoV-2 IgG antibodies. This was performed following the manufacturer’s instructions as reported previously [5]. The sensitivity and specificity of the assay as independently validated are 91.1% [*95% CI* 88.7 to 93.2] and 98.6% [97.4 to 99.4], respectively. Specimens giving a ratio of < 0.8 were reported as negative for this assay, a ratio of ≥ 1.1 were reported as positive, and a ratio between 0.8 and < 1.1 were reported as borderline.

### SARS-CoV-2 pseudovirus neutralisation assay

Samples were pre-screened using an in-house SARS-CoV-2 full-length spike ELISA [16], and only samples positive for binding antibodies were screened for neutralisation. SARS-CoV-2-pseudotyped lentiviruses were prepared by co-transfecting the HEK 293T cell line with either the SARS-CoV-2 original spike (D614G) or the SARS-CoV-2 beta spike (L18F, D80A, D215G, K417N, E484K, N501Y, D614G, A701V, 242-244 del) plasmids in conjunction with a firefly luciferase encoding pNL4 lentivirus backbone plasmid. For the neutralisation assay, heat-inactivated seropositive serum samples from blood donors were incubated with the SARS-CoV-2-pseudotyped virus for 1 h at 37 °C, 5% CO_2_. Subsequently, 1 × 10^4^ HEK 293T cells engineered to overexpress ACE-2 were added and incubated at 37 °C, 5% CO_2_, for 72 h upon which the luminescence of the luciferase gene was measured.

### Statistical analysis

**Fig. 1:**
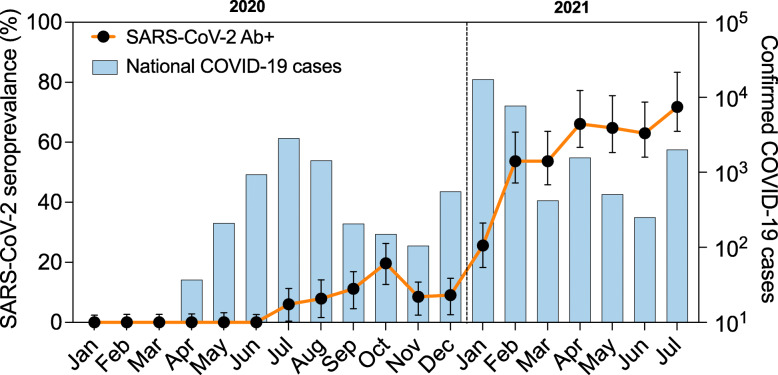
Overall SARS-CoV-2-adjusted seroprevalence over time superimposed on Malawi national PCR-confirmed COVID-19 cases. Black dots (together with *95% CI*) are estimated seroprevalence at each time point (month), adjusted for assay sensitivity and specificity. The histograms represent confirmed national COVID-19 cases per month, including asymptomatic and symptomatic cases. The vertical dotted line defines the transition from 2020 to 2021. SARS-CoV-2, severe acute respiratory syndrome coronavirus 2; COVID-19, coronavirus disease 2019; Ab+, positive for the detection of anti-SARS-CoV-2 receptor-binding domain (RBD) antibody

**Fig. 2:**
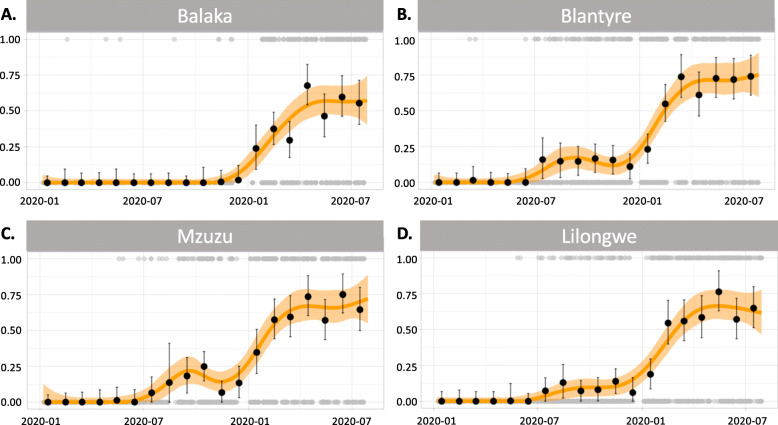
Flexible seroprevalence models. The model fits provide a smoothed estimate of the seroprevalence over time (January 2020 to July 2021) for each location. **A** Balaka. **B** Blantyre. **C** Mzuzu. **D** Lilongwe. The orange line is the line of best fit for the empirical data, using a smooth generalised additive model, along with light orange shading indicating *95% CI*. Black dots (together with *95% CI*) are estimated seroprevalence at each time point (months), adjusted for assay sensitivity and specificity. Grey dots (top and bottom of figures) show the individual-level data, which are either positive (1) or negative (0) for the detection of anti-SARS-CoV-2 receptor-binding domain (RBD) antibody

We performed statistical analyses and graphical presentations using the R statistical package, version 4.1.0, and GraphPad Prism v9.1.0 (GraphPad Software, LLC). The seroprevalence of SARS-CoV-2 antibodies was adjusted for the independently reported assay sensitivity (97% [*95% CI* 83.3 to 99.4]) and specificity (98% [*95% CI* 91.3 to 99.3]) using the bootComb (v1.0.1) R package [17]. Generalised additive models were used to estimate the seroprevalence curves from Figs. 1 and 2. A multivariable logistic regression model was developed with sex, age group, location, and time as predictors, to investigate the demographic factors associated with SARS-CoV-2 antibody positivity. To obtain smooth regression curves, penalised thin-plate regression splines (as implemented in the R package mgcv v1.8-35, [18]) were used to model the effect of time given the non-linear trend in seroprevalence over time. Confidence bands for the model fits were obtained using the estimated standard errors and a normal approximation for the model national seroprevalence but were derived using bootstrapping for the models stratified by location due to the lower sample sizes. Effects were considered statistically significant when the *p* value was less than 0.05.

To compute population weights, we first computed the proportions of participants by sex and 5-year age bands (15–19 up to 60–64) for each location and each month (e.g. proportion of males aged 20–24 in Blantyre for the month of August 2020). We then used the official Malawi census projection figures from the National Statistics Office [19] for the whole of Malawi for the mid-year points of 2019, 2020, and 2021 and linearly interpolated the numbers of Malawians of a given sex and 5-year age bands from January 2020 to July 2021 (the study period). We then calculated the same proportions of individuals in each sex and 5-year age band for every month for the census projection data (e.g. proportion of males aged 20–24 in August 2020). Finally, the population weights are obtained by computing the ratio of census projection proportions and the MBTS data proportions (Additional File 1: Table S1). The population weights were used to weigh the seroprevalence estimates and to fit the seroprevalence models.

## Results

### Participant demographics

A total of 5085 blood donor serum samples were selected from the four regional blood transfusion centres that were collected between January 2020 and July 2021. These included 1208 from Mzuzu (northern region), 1328 from Lilongwe (central region), 1198 from Balaka (eastern region), and 1351 from Blantyre (southern region) (Table 1). Compared with the 2018 Malawi Population and Housing Census [14], by the nature of the demographic of blood donors in Malawi, participants were more commonly male (72.6% in our study versus 48.5% in the census) and had more persons aged 20 to 49 years (80.1% versus 35.4%).

**Table 1:** Participant demographics and site characteristics

	Balaka	Blantyre	Lilongwe	Mzuzu	Overall
Region	Eastern	Southern	Central	Northern	–
Rural/urban	Rural	Urban	Urban	Urban	–
Pop density (persons/km^2^)^j^	205	3334	2455	1516	–
Sampling frame	All locations	Static	Static	Static	–
Gender, % male (*N*)	68.9% (825)	77.6% (1049)	64.5% (856)	79.6% (962)	72.6% (3692)
Age, median (*IQR*)	20.8 (19.7–24.9)	26.9 (21.9–34.1)	30.1 (23.8–37.9)	24.6 (20.2–31.6)	25.0 (20.7–33.2)
Sample size	1198	1351	1328	1208	5085

*IQR* interquartile range, *km* kilometre

^j^Source: 2018 Malawi Population and Housing Census main report

### Overall SARS-CoV-2 antibody seroprevalence

Of the 5085 samples, 1380 were positive for anti-SARS-CoV-2 RBD total antibody and 32 were borderline. After re-running the borderline samples using the separate commercial confirmatory ELISA as stated in the methods, 21 of the 32 were positive for anti-SARS-CoV-2 S2 and N IgG antibody, resulting in a total of 1401 anti-SARS-CoV-2 antibody-positive samples.

As part of quality control, we randomly selected 76 samples that were positive on the primary SARS-CoV-2 ELISA and retested them using the second ELISA targeting S2 and NP IgG. Seventy-one of the 76 samples were positive on the second ELISA, representing a concordance of 93.4% [*95% CI* 85.3 to 97.8]. We also used a recently published in-house ELISA [16] that targets binding antibodies against RBD of the original SARS-CoV-2 variant on a subset of 36 serum samples selected from the seroprevalence peaks. The concordance between the Wantai and in-house ELISA was 100%.

In an unweighted multivariable logistic regression model, the proportion of seropositive samples did not vary significantly by sex (*p* = 0.56) or age (*p* > 0.10 for all age groups using those 15–19 years old as a reference) but did vary geographically (*p* < 0.001 for all locations using Balaka as a reference) (Table 2). Amongst the urban areas, Lilongwe had lower seroprevalence than Mzuzu and Blantyre. As of July 2021, the overall population-weighted and assay characteristics (sensitivity and specificity)-adjusted seroprevalence was 70.2% (*95% CI* 62.2 to 81.6%) (Fig. 1).

**Table 2:** SARS-CoV-2 anti-RBD total antibody seroprevalence in blood donors by participant characteristics and location

Variable	Category	Sample size	Seropositive (frequency)	Unadjusted (%)	Estimate	SE	***z*** value	***p*** value
**Intercept**					− 6.12	0.76	− 8.11	< 0.001
**Gender**	Female	1393	407	29.2%	Ref	Ref	Ref	Ref
	Male	3692	994	26.9%	0.05	0.09	0.58	0.56
**Age group**	15–19^§^	912	265	29.1%	Ref	Ref	Ref	Ref
	20–29	2465	684	27.7%	− 0.04	0.11	− 0.32	0.75
	30–39	1068	284	26.6%	− 0.01	0.13	− 0.06	0.95
	40–49	596	154	25.8%	0.16	0.15	1.04	0.3
	50+	44	14	31.8%	0.2	0.38	0.53	0.6
**Location**	Balaka^φ^	1198	254	21.2%	Ref	Ref	Ref	Ref
	Blantyre	1351	416	30.8%	0.77	0.13	5.92	< 0.001*
	Lilongwe	1328	345	26.0%	0.6	0.13	4.56	< 0.001*
	Mzuzu	1208	386	32.0%	0.86	0.13	6.41	< 0.001*

This is an unweighted multivariable logistic regression model with sex, age group, location, and time as predictors

^§^Recruitment limited to blood donors ≥15 years of age

*Significant based on *p* value < 0.05

^φ^Balaka was used as a reference as it is the only Malawi Blood Transfusion Service (MBTS) static site that directly covers the rural area

### Temporal trend of SARS-CoV-2 seroprevalence

**Fig. 3:**
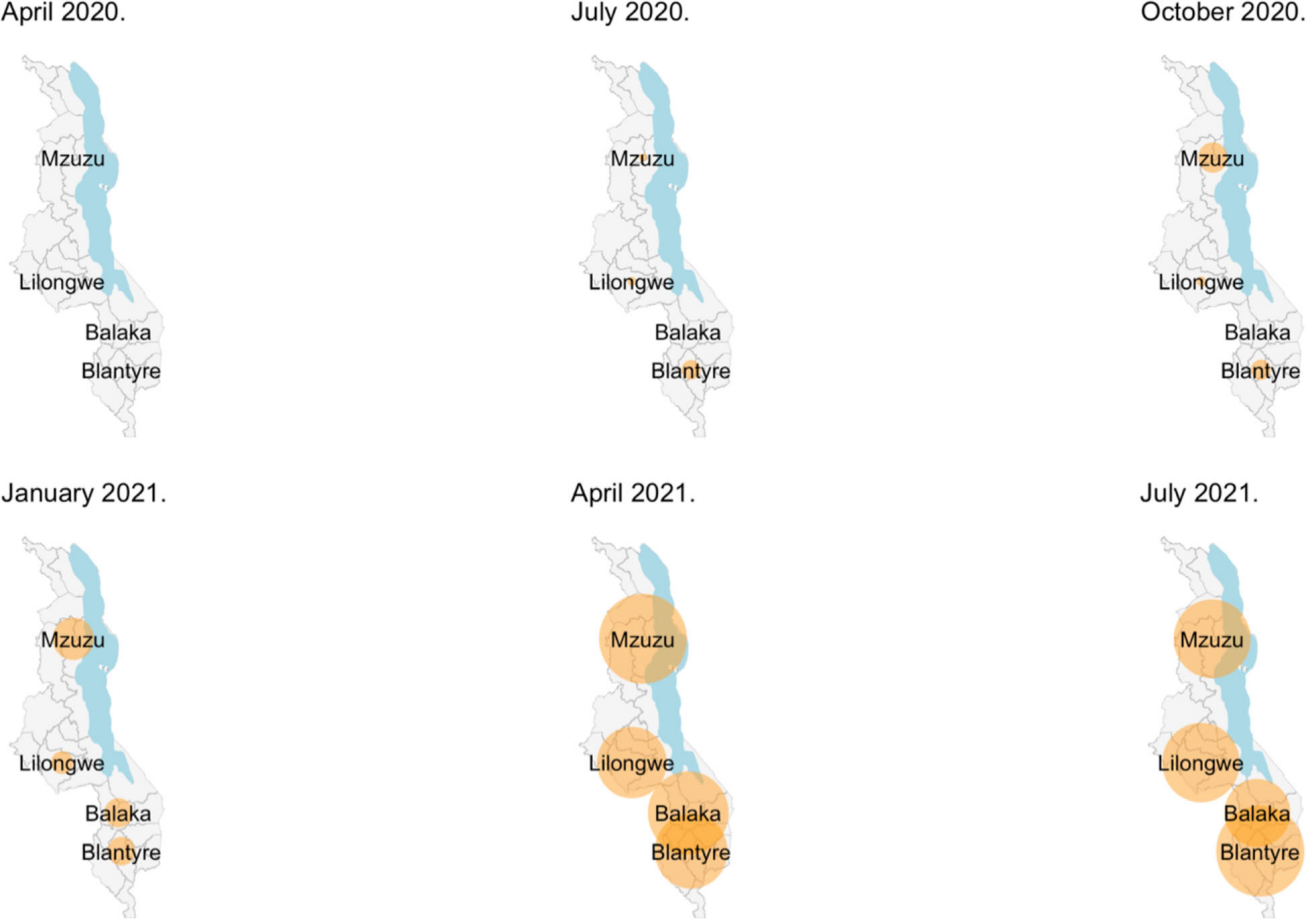
Seroprevalence bubble plot for the overall seroprevalence at three snapshots in time. The size of the bubble is proportional to the prevalence of samples positive for anti-SARS-CoV-2 receptor-binding domain (RBD) antibody within each location

The first PCR-confirmed case of COVID-19 identified by Malawi’s national surveillance system was on 2 April 2020, with the first peak of the national COVID-19 cases being July 2020 (2813 cases) and subsequently a second peak in January 2021 (17,380 cases) (Fig. 1). In this study, the first seropositive samples were observed in February 2020 in Balaka (1 sample) and March 2020 in Blantyre (2 samples) (Fig. 2). Population-weighted and assay characteristic-adjusted seroprevalence estimates increased with time, with two distinct waves that followed the same temporal trend as the reported national COVID-19 cases (Fig. 1). When aggregating serum samples from all locations, population-weighted assay characteristic-adjusted seropositivity was highest in October 2020 (18.5%), May 2021 (64.9%), and July 2021 70.2%) reflecting the two epidemic waves and the beginning of a third epidemic wave (Fig. 1). However, there were differences in the temporal trend in seroprevalence according to location (Fig. 2). Balaka had a 0% weighted and adjusted seroprevalence from January to October 2020, subsequently increasing from 3.8 to 63.5% between December 2020 and July 2021, peaking at 66.2% in April 2021. The peak weighted and adjusted seroprevalence in the first wave was October 2020 in Blantyre (20.6%), November 2020 in Lilongwe (13.1%), and October 2020 in Mzuzu (25.2%). Seroprevalence bubble plots at six periods in time demonstrated widespread exposure amongst blood donors across the country from January 2021 compared to the earlier periods (Fig. 3).

### Estimating predominant SARS-CoV-2 variants driving seroprevalence

**Fig. 4:**
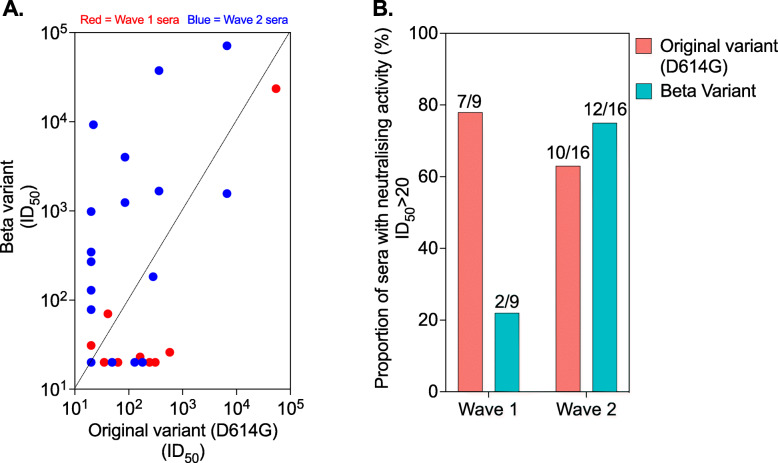
Neutralisation of viral variants using the sera from the first and second epidemic waves. Anti-RBD seropositive sera from the SARS-CoV-2 epidemic waves 1 and 2 were subjected to a SARS-CoV-2 pseudovirus neutralisation assay. **A** Correlation of SARS-CoV-2 antibody neutralisation potency against D614G WT and beta variant. **B** Proportion of wave 1 or wave 2 sera with neutralisation activity against D614G WT or beta variant. The threshold of detection for the neutralisation assay is *ID*_50_ > 20. RBD, receptor-binding domain

Sequencing data from Malawi deposited on Global Initiative on Sharing Avian Influenza Data (GISAID) show that the beta variant was the most dominant variant (96% [152/159]) identified from the samples collected in January 2021 [20], suggesting that Malawi’s second wave was driven by this variant. Following previous observations that antibodies elicited by the original variant (D614G WT) are less potent against the beta variant [16, 21], we reasoned that differential neutralisation potency by SARS-CoV-2 antibodies could be used to estimate the predominance of specific variants driving seroprevalence. We therefore measured pseudovirus neutralisation against the original variant and the beta variant in randomly selected seropositive sera collected from June to October 2020 (wave 1) compared to those collected in February 2021 (wave 2). Wave 1 sera were more potent against the original variant than the beta variant, while wave 2 sera were relatively more potent against the beta variant than the original variant (Fig. 4A, B). About 78% (7/9) of the wave 1 sera had neutralising activity against the original variant, but only 22% (2/9) of the wave 1 sera returned neutralising activity against the beta variant (Fig. 4B), while 75% (12/16) of the wave 2 sera had neutralising activity against the beta variant and 63% (10/16) of the wave 2 sera returned neutralising activity against the original variant (Fig. 4B). Furthermore, 19% (6/31) of the wave 2 sera showed no neutralisation against the beta variant or the original variant (Fig. 4A). Collectively, these results support the existing genomic sequencing evidence that Malawi’s second SARS-CoV-2 epidemic wave was driven by the beta variant and also indicates potential poor cross-protection from wave 1 infection (original variant) on wave 2 infection (beta variant).

## Discussion

Our study provides important insights into the dynamics of the COVID-19 epidemic in both urban and rural Malawi, with relevance to the southern African region. The seroprevalence estimates from this serosurvey are very high compared to the reported national surveillance figures for confirmed asymptomatic and symptomatic cases, but they do reflect the emergence and magnitude of the first and second COVID-19 epidemic waves in Malawi. They also provide evidence against the speculation that SARS-CoV-2 had been circulating more widely in Malawi before the first detected case in April 2020 or that circulation of endemic coronaviruses [9] had generated cross-reactive antibody responses. Data from pseudovirus neutralisation assays suggests that the second wave was predominantly driven by the beta variant and highlights poor cross-protection from the first wave on the second wave.

We report a high seroprevalence of 51.5% nationally in February 2021, mirroring the upsurge in confirmed COVID-19 cases, hospitalisations, and death in Malawi experienced during this period [1, 4]. This is consistent with a study in South African blood donors that reported seroprevalence rates ranging from 31.8 to 62.5% in January 2021 [22]. So far, the epidemic trajectory in Malawi as determined by the national confirmed COVID-19 cases, deaths, and hospitalisations has paralleled the epidemic trajectory in South Africa [1]. This is likely due to frequent human migration between the two countries, specifically in the last 12 months during which there has been an influx of groups of returning residents from South Africa to Malawi. This movement of people coincided with rapid surges in COVID-19 cases in the first and second epidemic waves in Malawi [23]. These findings support the need for a consolidated and standardised regional public health effort in Southern Africa to effectively reduce the risk of further epidemic waves across the region.

Genomic surveillance data from Malawi and South Africa suggests that this high seroprevalence in the second wave is, at least in part, driven by the emergence of the beta variant [20]. In agreement, our neutralisation data showed that a higher proportion of sera collected from the second wave (February 2021) had neutralising activity against the beta variant than sera from the first wave (June to October 2020), indicating antibody responses driven by infection with the beta variant rather than the original variant (D614G WT). This is consistent with previous studies showing that homologous convalescent sera retain higher in vitro neutralisation potency than heterologous convalescent sera against the infecting virus [16, 21, 24, 25]. It is also noteworthy that 19% of the second wave sera that were positive for antibodies to the SARS-CoV-2 RBD showed no neutralisation against the beta variant or D614G WT, highlighting a disconnect between qualitative antibody detection and functional activity. This discordance is likely due to low anti-RBD antibody titres as they have been shown to be associated with poor in vitro neutralisation [21]. It could also be due to infections from other variants of interest/concern.

The temporal kinetics of the SARS-CoV-2 seroprevalence in this serosurvey suggests that the first epidemic wave was largely confined to urban areas. This differs from the second wave, which was more rapid not only in its growth but also in its geographic spread, as shown by the high seroprevalence even in the rural areas. This is consistent with national surveillance reports showing that confirmed COVID-19 cases in the first wave were primarily from the three major cities, Blantyre, Lilongwe, and Mzuzu, but in the second wave, there was an increase in reported cases in rural areas [26]. Considering that no vaccine was being administered in Malawi between December 2020 and 11 March 2021, the poor cross-protection of first wave sera on the beta variant could in part have contributed to the rapid emergence and spread of this variant in the population. On the other hand, in other settings, high seroprevalence is being driven by high vaccination coverage, but in Malawi, as of July 2021, the coverage was less than 2% of the eligible population; hence, it is highly unlikely that the high seroprevalence was driven by vaccination.

This study has considerable strengths, including consistent monthly sampling, use of well-validated assays, and national geographical representation. There are however some important limitations. First, inherent in using a blood donor sampling frame is that they are not representative of the general population. To mitigate this, the seroprevalence estimates were weighted for population structure based on the official Malawi 2018 Population and Housing Census projection data for the study period. However, we were unable to evaluate the association between the behaviour of blood donors and risk of acquisition to SARS-CoV-2 infection, as this may bias the measured results away from the true population seropositivity. Population-based serosurvey is the ideal, and these could be highly recommended for future studies. Second, in some individuals, SARS-CoV-2 antibodies wane over time to undetectable levels leading to false negatives, especially in those who had asymptomatic COVID-19 [27, 28]. Therefore, the seroprevalence estimates are likely an underestimate of the cumulative exposure within this sampled population. However, the sampling bias and waning of antibody levels are unlikely to substantially alter the temporal trends reported in this study.

## Conclusions

We report a dramatic rise in SARS-CoV-2 seroprevalence from 18.5% in October 2020 to 64.9% in May 2021 in healthy blood donors as Malawi experienced the first and second COVID-19 epidemic waves, likely driven initially by the original variant (D614G WT) and then the beta variant [29–31]. The dynamics of SARS-CoV-2 exposure will therefore need to be taken into account in the formulation of the COVID-19 vaccination policy in Malawi and across the region. We recommend that future studies should include an adequate sample size to assess neutralisation activity across a larger panel of variants of concern/interest as a surrogate of protective immunity at a population level.

## Supplementary Information

**Additional file 1: Table S1.** Population weights calculated from census data. We used the National Statistics Office (NSO) 2018 Malawi Population and Housing Census Population Projections 2018-2050 estimates for 2019, 2020 and 2021 to calculate post-stratification population weights (the projections are for the mid-year point and we linearly interpolate on a monthly basis). These weights take 5-year age group, sex and month of donation into account. Month of donation is included as the age group and sex distribution of the donations can change considerably from month to month. The age range in the MBTS data spans from 15 years to 63 years. For this reason we used 5-year age band from 15-19 up to 60-64. The total population size is calculated for these age bands only. The table shows the first 20 rows of the population weights used to compute the estimate of the overall seroprevalence and for weighting. The full table has 380 rows and is not shown here, but can be made available.

## Data Availability

All data generated or analysed during this study are available upon request. R code for seroprevalence estimation, population weighting, adjustments for assay sensitivity and specificity, regression models, and graphs is available on GitHub (https://github.com/gitMarcH/Covid19_MBTS_serology).

## Abbreviations

ELISA: Enzyme-linked immunosorbent assay
GISAID: Global Initiative on Sharing Avian Influenza Data
HIV: Human immunodeficiency virus
IgG: Immunoglobulin G
KUHeS: Kamuzu University of Health Sciences
MBTS: Malawi Blood Transfusion Services
MLW: Malawi-Liverpool-Wellcome Trust Clinical Research Programme
RBD: Receptor-binding domain
S2: SARS-CoV-2 spike 2
SARS-CoV-2: Severe acute respiratory syndrome coronavirus 2
TTI: Transfusion transmissible infections
UK: United Kingdom
WHO: World Health Organization
