# Supplementary material for: SARS-CoV-2 exposure in Malawian blood donors: an analysis of seroprevalence and variant dynamics between January 2020 and July 2021
Source: BMC Med. 2021 Nov 19;19:303. doi: 10.1186/s12916-021-02187-y (PMC8601780; doi:10.1186/s12916-021-02187-y)
Supplement: Supplementary file 1 — Additional file 1: Table S1. Population weights calculated from census data. We used the National Statistics Office (NSO) 2018 Malawi Population and Housing Census Population Projections 2018-2050 estimates for 2019, 2020 and 2021 to calculate post-stratification population weights (the projections are for the mid-year point and we linearly interpolate on a monthly basis). These weights take 5-year age group, sex and month of donation into account. Month of donation is included as the age group and sex distribution of the donations can change considerably from month to month. The age range in the MBTS data spans from 15 years to 63 years. For this reason we used 5-year age band from 15-19 up to 60-64. The total population size is calculated for these age bands only. The table shows the first 20 rows of the population weights used to compute the estimate of the overall seroprevalence and for weighting. The full table has 380 rows and is not shown here, but can be made available. [file 12916_2021_2187_MOESM1_ESM.docx]

| **Location** | **Age group** | **Proportion of age-sex group in MBTS data** | **Proportion of age-sex group in census projection** | **Population weight** |
| --- | --- | --- | --- | --- |
| Lilongwe | 35-39 | 0.12 | 0.0514 | 0.428 |
| Blantyre | 35-39 | 0.0196 | 0.0514 | 2.6196 |
| Mzuzu | 35-39 | 0.0339 | 0.0514 | 1.5153 |
| Balaka | 35-39 | 0 | 0.0514 | NA |
| Lilongwe | 40-44 | 0.02 | 0.0406 | 2.0308 |
| Blantyre | 40-44 | 0.0588 | 0.0406 | 0.6905 |
| Mzuzu | 40-44 | 0.0339 | 0.0406 | 1.1982 |
| Balaka | 40-44 | 0.0149 | 0.0406 | 2.7213 |
| Lilongwe | 20-24 | 0.12 | 0.0921 | 0.7672 |
| Blantyre | 20-24 | 0.0392 | 0.0921 | 2.3478 |
| Mzuzu | 20-24 | 0.0508 | 0.0921 | 1.8107 |
| Balaka | 20-24 | 0.0746 | 0.0921 | 1.2337 |
| Lilongwe | 25-29 | 0.08 | 0.0773 | 0.9665 |
| Blantyre | 25-29 | 0.0784 | 0.0773 | 0.9858 |
| Mzuzu | 25-29 | 0.0169 | 0.0773 | 4.5618 |
| Balaka | 25-29 | 0 | 0.0773 | NA |
| Lilongwe | 30-34 | 0.04 | 0.0632 | 1.5793 |
| Blantyre | 30-34 | 0.0392 | 0.0632 | 1.6108 |
| Mzuzu | 30-34 | 0 | 0.0632 | NA |
| Balaka | 30-34 | 0 | 0.0632 | NA |

**Supplementary Files**

**Additional file 1: Table S1 – Example population weights calculated from census data for females in the month of January 2020**
